# Proteomics Insights into the Gene Network of *cis*9, *trans*11-Conjugated Linoleic Acid Biosynthesis in Bovine Mammary Gland Epithelial Cells

**DOI:** 10.3390/ani12131718

**Published:** 2022-07-02

**Authors:** Liying Peng, Ge Bai, Chunzheng Wang, Jianan Dong, Yongjun Liu, Zhe Sun, Yuguo Zhen, Guixin Qin, Xuefeng Zhang, Natnael Demelash, Tao Wang

**Affiliations:** 1 JLAU-Borui Dairy Science and Technology R&D Center, Key Laboratory of Animal Nutrition and Feed Science of Jilin Province, Key Laboratory of Animal Production Product Quality and Security Ministry of Education, College of Animal Science and Technology, Jilin Agricultural University, Changchun 130118, China; ply759121@163.com (L.P.); baige1125@163.com (G.B.); 15944699758@163.com (C.W.); dong__jianan@163.com (J.D.); lyj8595@163.com (Y.L.); sunzhe198615@163.com (Z.S.); nickzhen@263.net (Y.Z.); qgx@jlau.edu.cn (G.Q.); zhangxuefengjlau@163.com (X.Z.); ednatnael@gmail.com (N.D.); 2 Changchun Institute of Biological Products Co., Ltd., Changchun 130012, China; 3 Ningxia Agricultural Reclamation Helan Mountain Dairy Co., Ltd., Yinchuan 750028, China; 4 Postdoctoral Scientific Research Workstation, Feed Engineering Technology Research Center of Jilin Province, Changchun Borui Science & Technology Co., Ltd., Changchun 130118, China; 5 College of Life Science, Jilin Agricultural University, Changchun 130118, China; 6 College of Agriculture and Environmental Science, Dilla University, Dilla P.O. Box 419, Ethiopia

**Keywords:** biosynthesis, bovine mammary gland, *cis*9, *trans*11-conjugated linoleic acid, energy metabolism pathways, MAC-T cells, stearoyl-CoA desaturase 1

## Abstract

**Simple Summary:**

*Cis*9, *trans*11-conjugated linoleic acid (c9, t11-CLA) is a bioactive compound that is mainly found in ruminant products and has a variety of beneficial biological functions in human health. However, the gene network of c9, t11-CLA biosynthesis in the mammary gland is still not well documented. Therefore, this study aimed to elucidate the stearoyl-coenzyme A desaturase (SCD1)-dependent gene network of c9, t11-CLA biosynthesis in MAC-T cells from the perspective of energy metabolism. The results showed that the deficiency of stearoyl-coenzyme A desaturase (SCD1), led by CAY10566, blocked the biosynthesis of c9, t11-CLA. In total, 60 SCD1-related proteins that were mainly involved in energy metabolism pathways were primary screened by Tandem mass tag-based quantitative proteomics analysis. Moreover, 17 proteins were validated under the parallel reaction monitoring analysis. Then, 11 genes involved in energy metabolism pathways were verified to have negative relationships with SCD1 after small RNA interference analysis. Based on the above results, we concluded that the 11 genes involved in energy metabolism pathways had an impact on the SCD1-dependent molecular mechanism of c9, t11-CLA biosynthesis. This study provided a fresh understanding of the gene network of c9, t11-CLA biosynthesis in the mammary gland from a metabolism perspective.

**Abstract:**

The objective of the study was to elucidate the stearoyl-coenzyme A desaturase (SCD1)-dependent gene network of c9, t11-CLA biosynthesis in MAC-T cells from an energy metabolism perspective. The cells were divided into the CAY group (firstly incubated with CAY10566, a chemical inhibitor of SCD1, then incubated with trans-11-octadecenoic acid, (TVA)), the TVA group (only TVA), and the control group (without CAY, TVA). The c9, t11-CLA, and TVA contents were determined by gas chromatography. The mRNA levels of SCD1 and candidate genes were analyzed via real-time PCR. Tandem mass tag (TMT)-based quantitative proteomics, bioinformatic analysis, parallel reaction monitoring (PRM), and small RNA interference were used to explore genes involved in the SCD1-dependent c9, t11-CLA biosynthesis. The results showed that the SCD1 deficiency led by CAY10566 blocked the biosynthesis of c9, t11-CLA. In total, 60 SCD1-related proteins mainly involved in energy metabolism pathways were primarily screened by TMT-based quantitative proteomics analysis. Moreover, 17 proteins were validated using PRM analysis. Then, 11 genes were verified to have negative relationships with SCD1 after the small RNA interference analysis. Based on the above results, we concluded that genes involved in energy metabolism pathways have an impact on the SCD1-dependent molecular mechanism of c9, t11-CLA biosynthesis.

## 1. Introduction

*Cis*9, *trans*11-conjugated linoleic acid (c9, t11-CLA) is a bioactive compound with a variety of beneficial biological functions, including an important role in improving the nutritional value of milk [1,2,3]. For dairy cows, the main source of c9, t11-CLA in milk is de novo biosynthesis in the mammary glands and the remaining portion is synthesized by rumen microorganisms [4,5,6]. The de novo biosynthesis of c9, t11-CLA in the mammary glands refers to the c9, t11-CLA dehydrogenated from trans-11 vaccenic acid (TVA) by stearoyl-coenzyme A desaturase (SCD1) [7,8,9]. TVA is synthesized by rumen microorganisms and is subsequently absorbed and transported to the mammary tissue for c9, t11-CLA biosynthesis. The rumen microbial synthesis of c9, t11-CLA refers to the transformation of dietary linoleic acid into c9, t11-CLA under the isomerization by microorganisms in the rumen [10]. TVA and SCD1 are two essential components of the biosynthesis process of c9, t11-CLA. To date, five SCD isoforms have been discovered [11]. SCD1 is located in the endoplasmic reticulum and mainly expressed in the mammary glands, liver, and adipose tissue [12,13]. SCD1 is crucial for the c9, t11-CLA content in milk [14,15,16,17]. Factors that regulate the SCD1 activity or the synthesis of TVA in rumen can alter the milk content of *cis*9, *trans*11-CLA [4]. However, the SCD1-dependent gene network of c9, t11-CLA biosynthesis in mammary gland is still not well elucidated. It is important for more studies to examine the molecular mechanism of c9, t11-CLA synthesis in order to reveal the individual difference in c9, t11-CLA synthesis ability of dairy cows. This seems to be a potential option that would improve the nutritive value of milk.

It is well known that pentose phosphate pathway (PPP) is one of the ubiquitous catabolic pathways of glucose, and the reductase II (NADPH) produced in PPP is important for the desaturation of fatty acids [18]. Therefore, we proposed a hypothesis that the genes involved in energy metabolism pathways may have an impact on the SCD1-dependent molecular mechanism of c9, t11-CLA biosynthesis. The objective of the study was to elucidate the SCD1-dependent gene network of c9, t11-CLA biosynthesis from the perspective of energy metabolism. MAC-T cells, an immortalized bovine mammary gland epithelial cell line, were used in this study [19,20,21]. The cells were divided into the CAY group (firstly incubated with CAY10566, a chemical inhibitor of SCD1 then incubated with trans-11-octadecenoic acid (TVA)), the TVA group (only TVA), and control group (without CAY, TVA). The c9, t11-CLA and TVA contents were determined by gas chromatography. The mRNA levels of SCD1 and candidate genes were analyzed using real-time PCR. Tandem mass tag (TMT)-based quantitative proteomics, bioinformatic analysis, parallel reaction monitoring (PRM), and small RNA interference were used to explore the genes involved in the c9, t11-CLA biosynthesis. This study may provide a fresh understanding of the SCD1-dependent gene network of c9, t11-CLA biosynthesis in the mammary gland.

## 2. Materials and Methods

### 2.1. Materials

Total RNA was extracted from MAC-T cells and complementary DNA (cDNA) was generated using commercial kits (TIANGEN Biotech Co., Ltd., Beijing, China). The kit for real-time polymerase chain reaction (real-time PCR) was from Foregene Co., Ltd., Beijing, China. CAY10566 (Cayman Chemical Company, Ann Arbor, MI, USA), a chemical inhibitor of SCD1, was used for the construction of SCD1-inhibited MAC-T cells model [20]. Trans-11-octadecenoic acid (Sigma-Aldrich, St. Louis, MO, USA), fatty acid standard 37 component FAME mix (FAME37), and trans-11-octadecenoic methyl ester (NU-CHEKPREP, Elysian, MN, USA), and methyl 9(Z), 11(E)-octadecadienoate (Matreya LLC, State College, PA, USA) were purchased for the fatty acids’ analysis.

### 2.2. Cell Treatment and Real-Time PCR Analysis

MAC-T cells were maintained in DMEM/High glucose medium, and the culture conditions were similar to those described previously [22]. MAC-T cells were divided into three groups: CAY group (incubated with 50 µM TVA for 2.5 h after the treatment of CAY10566 at concentrations of 10 nM for 12 h), which was considered as the SCD1-inhibited MAC-T cells model, TVA group (only incubated with 50 µM TVA for 2.5 h), and control group (without CAY, TVA). CAY10566 was proved to inhibit SCD1 at the transcriptional level in our previous works [20,23]. RNA was reverse transcribed after measuring the concentration using a NanoDrop 2000 ultra-micro spectrophotometer (Thermo Fisher Scientific, Waltham, MA, USA). After reverse transcription, the final assay was carried out in the real-time fluorescence quantification system (Bio-Rad, Hercules, CA, USA). The primer sequences are shown in Supplementary Table S1 [23]. The β-actin was used for a threshold comparison with the target gene for final relative quantification [24].

### 2.3. Fatty Acids Analysis

The total lipids of the cells in those three groups (CAY group, TVA group, control group; three replicates in each group) were extracted after adding chloroform/methanol (2:1, *v*/*v*), and the specific steps were as follows [25,26,27]: 10 mL of chloroform/methanol was added to the cells, shaking and ultrasound were applied to break the cells for lipid extraction, and excess pure water was added to wash the cells. Subsequently, a centrifugation at 2500× *g* for 5 min was performed to take the organic layer; 10 mol/L KOH solution and methanol solution were added for methyl esterification, after which 12 mol/L H_2_SO_4_ was added to samples for methyl esterification. Finally, hexane (3 mL) was added, followed by centrifugation for 2000× *g* for 10 min. The supernatant (fatty-acid methyl esters (FAMEs)) was subjected to gas chromatography (GC) analysis. FAMEs were analyzed using an Agilent 7890B GC system (Agilent Technologies, Santa Clara, CA, USA) equipped with a DB-Fast FAME fused silica capillary column (90 m × 0.32 mm × 0.2 µm). Front inlet and FID temperatures were maintained at 240 °C and 260 °C, respectively. The hydrogen flow rate to the detector was 30 mL/min, and the airflow rate was 400 mL/min. The split ratio was 10:1 [28,29]. Percentages of individual fatty acids were calculated as the ratio of individual GC areas to the total area of identified fatty acids [27].

### 2.4. Identification of Differentially Expressed Proteins by Tandem Mass Tag (TMT)-Based Quantitative Proteomics

The protein samples of those three groups (CAY group, TVA group, control group; three replicates in each group) were prepared as follows: a 4-fold volume of lysis buffer (8 mol/L urea, 1% protease inhibitor) was used to crack the cells [30]. To obtain the protein sample, the supernatant was collected through centrifuging at 12,000× *g* for 10 min at 4 °C then the protein concentration was measured using a BCA kit, taking an equal amount of protein for enzyme digestion. TCA was slowly added to the sample, then mixed to precipitate for 2 h at 4 °C. The sample was centrifuged at 4500× *g* for 5 min, and the precipitate was washed with pre-cooled acetone after discarding the supernatant. Dried, TEAB was then added after the precipitate was dried and broken up by sonication and then trypsin was added overnight at a ratio of 1:50 (protease:protein). Dithiothreitol (DTT) and iodoacetamide (IAA) were individually added to a final concentration of 5 mM and 11 mM, respectively [31,32]. The trypsinized peptides were desalted using a Strata X C18 column and freeze-dried [33].

Then, the peptides were dissolved in 0.5M TEAB, and the peptide was labeled according to the instructions of the tandem mass tags (TMT) kit. The peptides were graded by high-pH, reversed-phase HPLC using an Agilent 300 Extend column C18 (5 μm × 4.6 mm × 250 mm), separated combined, and then freeze-dried under vacuum for subsequent usage [31,34]. The peptides were dissolved in liquid chromatography mobile phase A (aqueous solution of 0.1% HCOOH + 2% acetonitrile) and separated using an ultra-performance liquid chromatography system. Mobile phase B contained 0.1% HCOOH and 90% acetonitrile [35,36]. The liquid phase gradient was set as follows: 0–38 min, 6–22% B; 38–52 min, 22–32% B; 52–56 min, 32–80% B; 56–60 min, 80% B. Flow rate was maintained at 500 nL/min; ion source voltage: 2.1 kV, primary mass spectrometry scan range set to 350–1600 m/z and the scan resolution was set to 120,000; the secondary mass spectrometry scan range was fixed at 100 m/z and the secondary scan resolution was set to 30,000 [37]. The mass spectrometry proteomics data were deposited to the ProteomeXchange Consortium via the PRIDE [38] partner repository with the dataset identifier PXD028620.

### 2.5. Protein Functional Annotation and Bioinformatic Analysis

Gene ontology (GO) was used to describe the properties of genes and gene products in living organisms, and was divided into biological process, cellular component, and molecular function [39,40]. GO annotation analysis was performed using the UniProt-GOA database (http://www.ebi.ac.uk/GOA/, accessed on 28 December 2020) and the protein sequence-based algorithm software InterProScan. The protein structural domain annotation was performed using the InterProScan software and the corresponding InterPro structural domain database (http://www.ebi.ac.uk/interpro/, accessed on 28 December 2020) for the protein domain annotation of the identified proteins. KEGG pathway annotation was performed on the submitted proteins using the online service tool KAAS.

### 2.6. Parallel Reaction Monitoring (PRM) Validation

To assess the reliability of proteomic differential protein data, parallel reaction monitoring (PRM) was used to verify the differential proteins related to SCD1. PRM is a method used for the selective detection and quantification of target proteins and peptides using ion monitoring technology [41,42].

### 2.7. Small RNA Interference

MAC-T cells were transfected with specific siRNA for bovine 6-phosphogluconolactonase (PGLS), phosphofructokinase, liver type (PFKL), aldolase, fructose-bisphosphate A (ALDOA), triosephosphate isomerase 1 (TPI1), glyceraldehyde-3-phosphate dehydrogenase (GAPDH), phosphoglycerate kinase 1 (PGK1), phosphoglycerate mutase 1 (PGAM1), enolase 1 (ENO1), pyruvate kinase M1/2 (PKM), lactate dehydrogenase A (LDHA), and lactate dehydrogenase B (LDHB), or a scrambled siRNA using Lipofectamine^®^ RNAIMAZ Reagent (Invitrogen, CA, USA). The specific siRNAs are shown in Supplementary Table S2 [20]. After 24 h, the cells were incubated with 50 µM trans-11 C18:1 for 2.5 h. Then, cells were harvested for total mRNA extraction and real-time PCR analysis [20]. The reaction system of real-time PCR and the fluorescence quantitative reaction procedure are listed in Supplementary Tables S3 and S4.

### 2.8. Statistical Analysis

All data are presented as the mean ± standard error (S.E.). The data of mRNA relative expression, TVA and c9, t11-CLA level were analyzed with the one-way analysis of variance (one-way ANOVA, SPSS 24.0, SPSS Inc., Chicago, IL, USA). Differences were considered significant at *p* < 0.05, highly significant at *p* < 0.01. Differential proteins were screened from quantifiable proteins with *p*-value < 0.05, and a differential expression change more than 1.3/1 was defined as the threshold of change for significant up-regulation, and a change of less than 1/1.3 as the threshold of change for significant down-regulation. TMT labeling and PRM verification results were compared using the GraphPad Prism 8.0.2 (GraphPad Software, San Diego, CA, USA).

## 3. Results

### 3.1. Effects of CAY and TVA on SCD1 mRNA Expression

**Figure 1:**
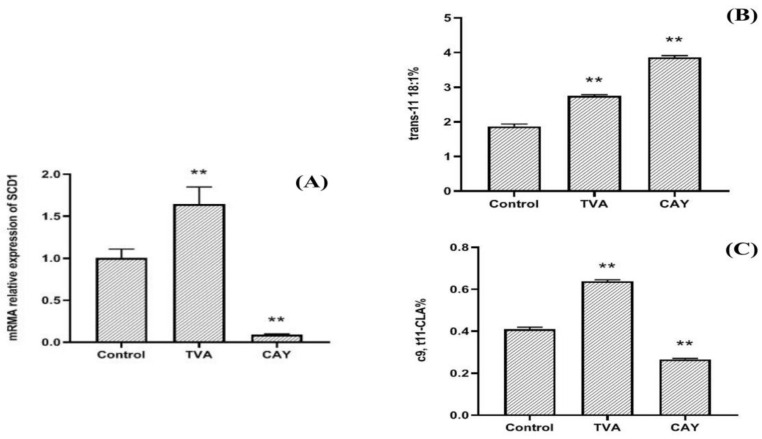
The SCD1 mRNA relative expression (**A**); TVA accumulation (**B**); % of total identified fatty acids) and c9, t11-CLA synthesis (**C**); % of total identified fatty acids in MAC-T cells under different incubation conditions. CAY group (SCD1-inhibited MAC-T cells model): incubated with 50 μM TVA for 2.5 h after CAY10566 treatment at concentrations of 10 nM for 12 h, TVA group: only incubated with 50 μM TVA for 2.5 h, and control group: without CAY, TVA. ** *p* < 0.01.

The results (Figure 1A) indicated that compared with the control and TVA group, the mRNA relative expression of SCD1 was significantly decreased after CAY treatment (*p* < 0.01), and the inhibitory effect was about 90%. After adding TVA, SCD1 activity was promoted, and its mRNA expression was significantly higher than that in the control group (*p* < 0.05).

### 3.2. Effects of CAY and TVA on TVA Accumulation and c9, t11-CLA Synthesis

After the addition of TVA and CAY, TVA accumulation in the CAY group was significantly higher than that in the TVA group (Figure 1B; *p* < 0.01), while the c9, t11-CLA biosynthesis was significantly lower than that in the TVA group (Figure 1C; *p* < 0.01).

### 3.3. Identification of Differentially Expressed Proteins

**Figure 2:**
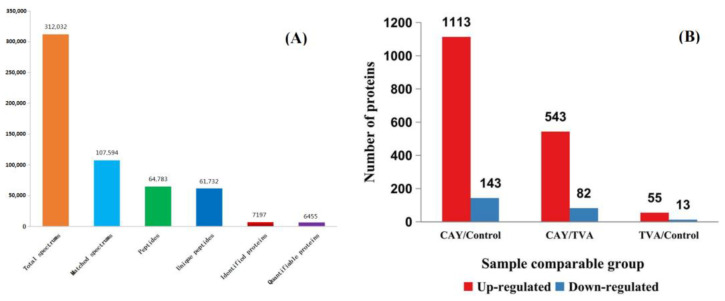
Statistics of mass spectrum data (**A**) and the number of differentially expressed proteins difference protein between groups of MAC-T cells under different incubation conditions (**B**). CAY group (SCD1-inhibited MAC-T cells model): incubated with 50 μM TVA for 2.5 h after CAY10566 treatment at concentrations of 10 nM for 12 h, TVA group: only incubated with 50 μM TVA for 2.5 h, and control group: without CAY, TVA. Differential proteins were screened from quantifiable proteins with *p*-value < 0.05, and a differential expression change more than 1.3/1 was defined as the threshold of change for significant up-regulation, while a change of less than 1/1.3 was the threshold of change for significant down-regulation.

**Figure 3:**
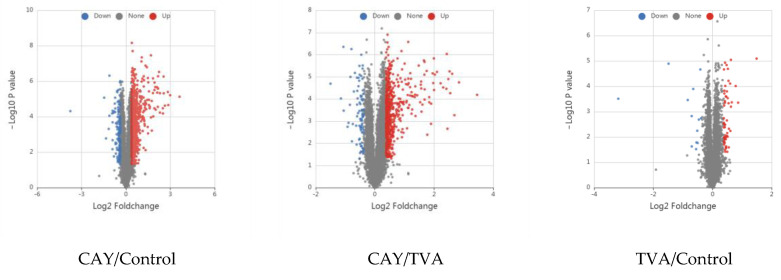
Volcanic map of differentially expressed proteins in MAC-T cells under different incubation conditions. The horizontal coordinate indicates the fold change (log2 transformation), while the vertical coordinate indicates the *p*-values (log10 transformation). Red dots indicate upregulation and blue dots indicate downregulation.

A total of 312,032 secondary spectra were generated using mass spectrometry analysis of the obtained protein samples and 6455 quantifiable proteins were obtained (Figure 2A). The two-way comparison between the control, TVA, and CAY groups regarding protein expression is summarized in Figure 2B. A volcanic map of these differentially expressed proteins (red dots indicate up-regulated proteins and blue dots indicate down-regulated proteins) in MAC-T cells under different incubation conditions is shown in Figure 3.

### 3.4. Functional Annotation of Differentially Expressed Proteins

**Figure 4:**
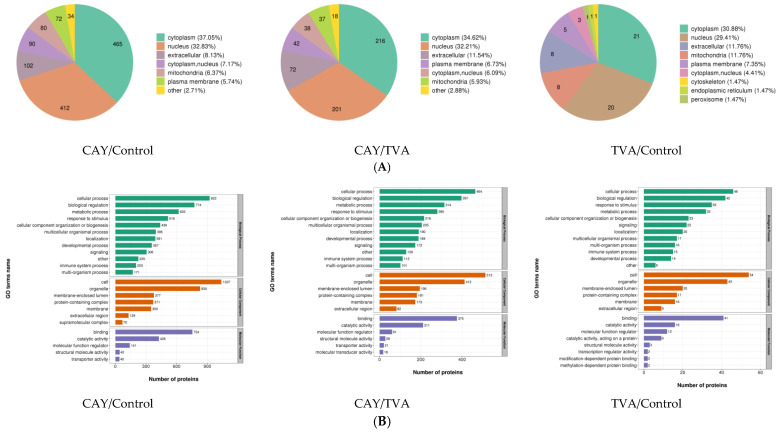
The subcellular structure localization of differentially expressed proteins (**A**) and GO secondary annotation (**B**) in MAC-T cells under different incubation conditions. CAY group (SCD1-inhibited MAC-T cells model): incubated with 50 μM TVA for 2.5 h after the treatment of CAY10566 at concentrations of 10 nM for 12 h, TVA group: only incubated with 50 μM TVA for 2.5 h, and control group: without CAY, TVA.

The differential proteins identified in each group were mainly located in the cytoplasm, nucleus, extracellular space, mitochondria, and cell membrane, with the highest number of proteins being localized in the cytoplasm and nucleus (Figure 4A). Differential proteins were found to be involved in a total of 28 terms of biological functions, mainly focusing on biological regulation, metabolic processes, binding, and catalytic activity (Figure 4B).

### 3.5. GO and KEGG Analysis of Differentially Expressed Proteins

**Figure 5:**
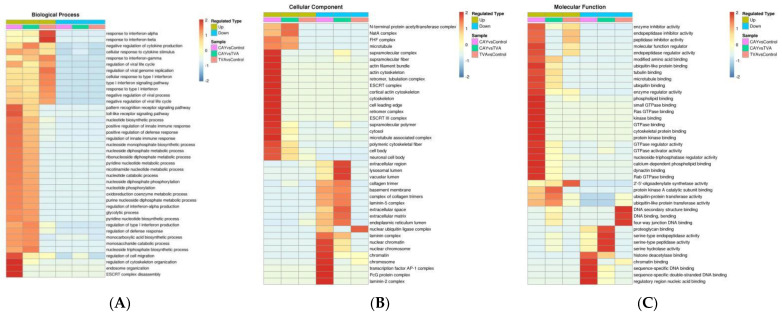
Cluster analysis heat map based on GO enrichment classifications: biological process (**A**); cellular component (**B**); molecular function (**C**). GO enrichment was performed for differential proteins in MAC-T cells under different incubation conditions, and cluster analysis was performed to determine the correlation between the functions of differential proteins. The horizontal direction indicates the enrichment test results of different groups, and the vertical direction shows a description of differential proteins’ enrichment-related functions. Different sets of differential proteins and color blocks correspond with the functional descriptions and indicate the degree of enrichment, where red indicates stronger enrichment (the deeper the red, the stronger the enrichment) and blue indicates weaker enrichment (the lighter the blue, the weaker the enrichment).

Following GO classification with biological process (BP), cellular component (CC), and molecular function (MF), a cluster analysis was conducted to compare the functional correlations between DE proteins in the experimental groups; the results are displayed as a heat map (Figure 5).

**Figure 6:**
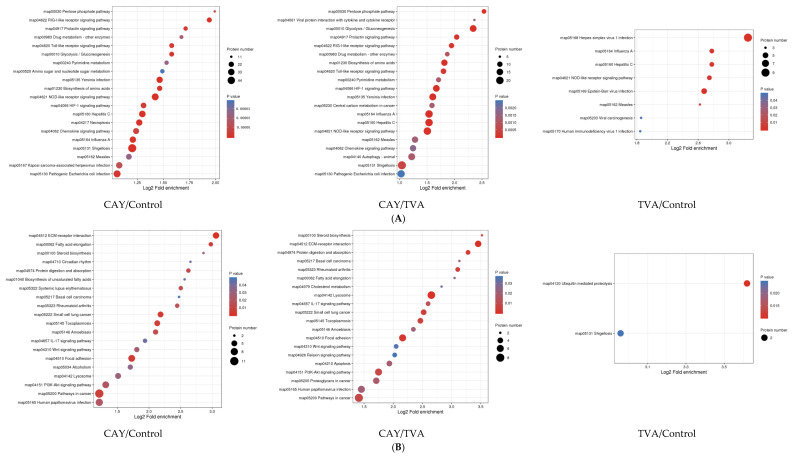
KEGG enrichment pathway map of the up-regulated proteins (**A**) and down-regulated proteins (**B**) in MAC-T cells under different incubation conditions. CAY group (SCD1-inhibited MAC-T cells model): incubated with 50 μM TVA for 2.5 h after the treatment of CAY10566 at concentrations of 10 nM for 12 h, TVA group: only incubated with 50 μM TVA for 2.5 h, and control group: without CAY, TVA.

The up-regulated and down-regulated differential proteins of the two comparison groups were enriched by about 30 terms under the three categories, and we mainly focused on the pathways related to energy metabolism. Among the secondary pathways, the glycolytic pathway and nucleotide catabolic pathway of the up-regulated proteins were found in the CAY/control group, and the functional pathways of lipid acyl coenzyme A biological processes, fatty acid elongase activity, and fatty acid synthase activity of the down-regulated proteins were associated with fatty acids; in the CAY/TVA group, the glycolytic pathway and nucleotide catabolic pathway were equally enriched in the up-regulated differential proteins, as well as in the glycolytic pathway and nucleotide catabolic pathway. Regarding the down-regulated differential protein enrichment pathways, lipoprotein catabolism and sphingolipid catabolism were associated with fatty acids. The enrichment process of the TVA/control group did not have the desired related pathways. KEGG pathway enrichment statistical analysis was performed for up-regulated and down-regulated differential proteins (Figure 6), similar to the GO analysis. Glycolysis, pentose phosphate, and amino acid and nucleotide energy metabolism pathways, fatty acid extension pathways, and unsaturated fatty acid biosynthesis pathways were enriched. In addition, we determined the common pathways from GO and KEGG analyses, and the results are shown in Supplementary Tables S5–S8.

### 3.6. PRM Validation

**Figure 7:**
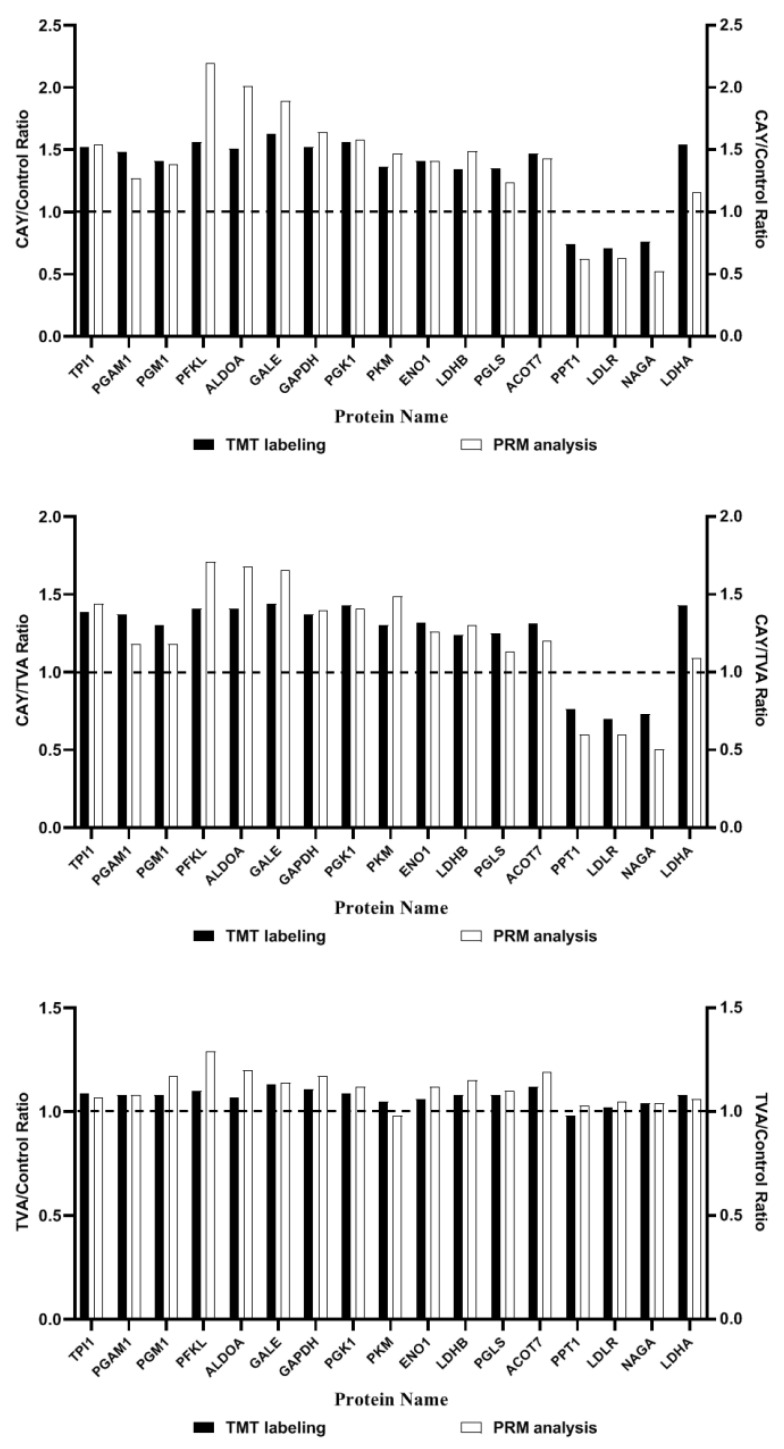
Tandem mass tag labeling and parallel reaction monitoring analysis among the differentially expressed proteins found in MAC-T cells under different incubation conditions. CAY group (SCD1-inhibited MAC-T cells model): incubated with 50 μM TVA for 2.5 h after the treatment of CAY10566 at concentrations of 10 nM for 12 h, TVA group: only incubated with 50 μM TVA for 2.5 h, and control group: without CAY, TVA.

Among the differential proteins screened from the proteomics analysis, 17 were selected for PRM validation, and a comparison between the verification results and the TMT labeling results is shown in Figure 7. The ploidy changes are based on 1; a score greater than 1 represents up-regulated proteins and a score of less than 1 represents down-regulated proteins. PRM validation results and proteomics trends were consistent among the groups, except for PKM and PPT1 in the TVA/control group, indicating the reliability of the results of this experiment.

### 3.7. The Responses of SCD1 after the Knockdown of PGLS, PFKL, ALDOA, TPI1, GAPDH, PGK1, PGAM1, ENO1, PKM, LDHA, and LDHB

**Figure 8:**
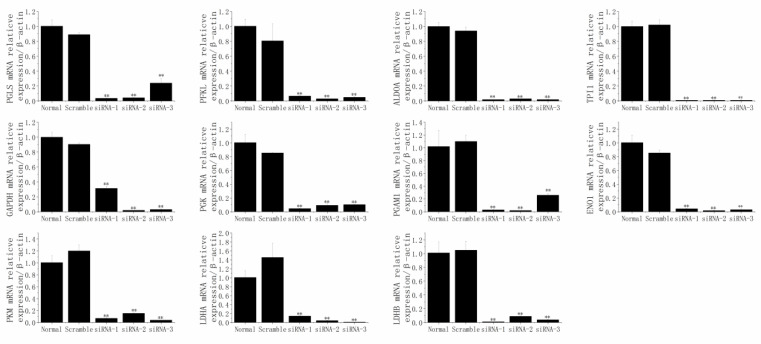
The mRNA relative expression levels of 6-phosphogluconolactonase (PGLS), phosphofructokinase, liver type (PFKL), aldolase, fructose-bisphosphate A (ALDOA), triosephosphate isomerase 1 (TPI1), glyceraldehyde-3-phosphate dehydrogenase (GAPDH), phosphoglycerate kinase 1 (PGK1), phosphoglycerate mutase 1 (PGAM1), enolase 1 (ENO1), pyruvate kinase M1/2 (PKM), lactate dehydrogenase A (LDHA), and lactate dehydrogenase B (LDHB) were successfully and individually reduced by their specific siRNA sequences in MAC-T cells. ** *p* < 0.01.

The results (Figure 8) indicated that PGLS, PFKL, ALDOA, TPI1, GAPDH, PGK1, PGAM1, ENO1, PKM, LDHA, and LDHB were successfully and individually reduced by their specific siRNA sequences (*p* < 0.01).

**Figure 9:**
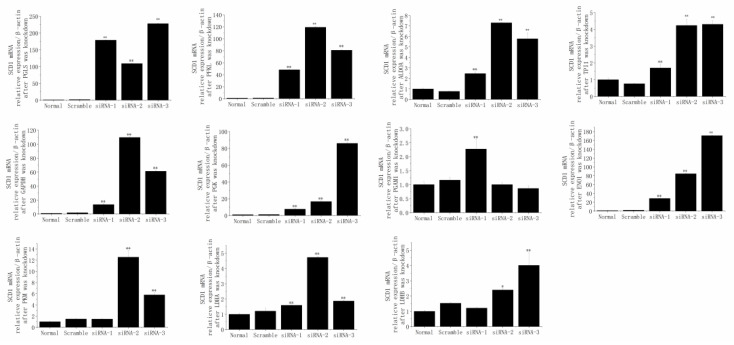
After individual PGLS, PFKL, ALDOA, TPI1, GAPDH, PGK1, PGAM1, ENO1, PKM, LDHA, and LDHB knockdown, the mRNA relative expression levels of SCD1 (stearoyl CoA desaturase 1) were altered in MAC-T cells. * *p* < 0.05, ** *p* < 0.01.

After the knockdown of PGLS (siRNA-1, siRNA-2, siRNA-3), PFKL (siRNA-1, siRNA-2, siRNA-3), ALDOA (siRNA-1, siRNA-2, siRNA-3), TPI1 (siRNA-1, siRNA-2, siRNA-3), GAPDH (siRNA-1, siRNA-2, siRNA-3), PGK (siRNA-1, siRNA-2, siRNA-3), PGAM1 (siRNA-1), ENO1 (siRNA-1, siRNA-2, siRNA-3), PKM (siRNA-2, siRNA-3), LDHA (siRNA-1, siRNA-2, siRNA-3), or LDHB (siRNA-2, siRNA-3), the mRNA expression of SCD1 was significantly elevated (*p* < 0.05; Figure 9).

**Figure 10:**
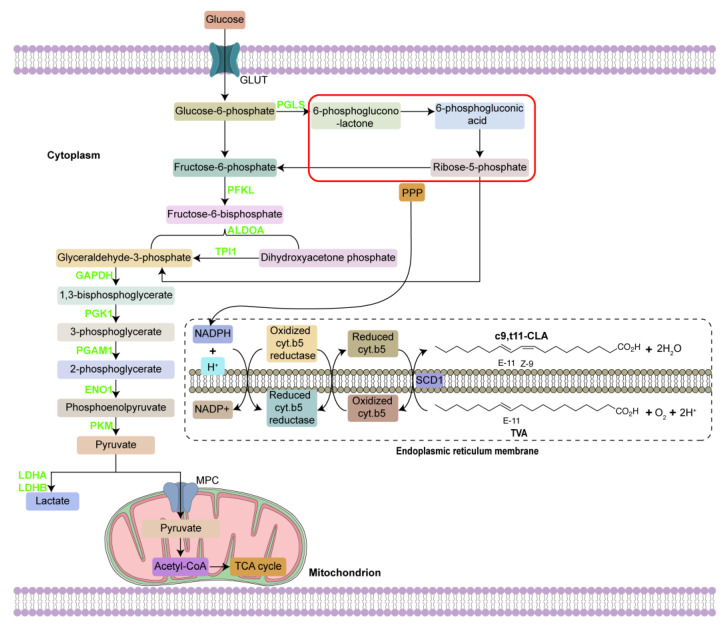
The SCD1-dependent gene network of c9, t11-CLA biosynthesis from the perspective of energy metabolism. The green color protein indicates that they were verified to have negative relationships with SCD1.

Therefore, based on the above data the SCD1-dependent gene network of c9, t11-CLA biosynthesis is outlined for the mammary gland in Figure 10, from the energy metabolism perspective.

## 4. Discussion

As a key enzyme in fatty acid biosynthesis, SCD1 is highly expressed in adipose tissue, liver, and especially in lactating mammary tissue, which plays an important role in the biosynthesis of monounsaturated fatty acids [43,44]. The inhibition of SCD1 expression causes a series of physiological changes in the body related to fatty acid oxidation, lipid biosynthesis, and triacylglycerol (TAG) deficiency [45,46]. In this study, the contents of c9, t11-CLA, and TVA were determined by gas chromatography after the incubation with CAY10566, while the mRNA levels of SCD1 were analyzed by real-time PCR. Our results showed that CAY10566 successfully inhibited SCD1, which could not catalyze the conversion of TVA to c9, t11-CLA and is consistent with a previous study by our group [23]. The MAC-T cells model with loss of SCD1 function was successfully constructed, and also showed that SCD1 plays a critical role in regulating the c9, t11-CLA content. Proteins are essential to cell activity, and their characterization is extremely important for revealing life phenomena and understanding life activities; therefore, research at the protein level is crucial [47]. SCD1 has been directly or indirectly confirmed in several studies to be a desaturase affecting the c9, t11-CLA content in dairy cows and goats, acting together with other proteins for fatty acid biosynthesis [5,15,25,48].

After verifying the function of SCD1 in regulating c9, 11-CLA, using more accurate and specific omics techniques to search SCD1-related proteins is necessary for the better understanding of the gene network of c9, t11-CLA biosynthesis in the mammary glands of dairy cows. Therefore, in this study, tandem mass tag (TMT)-based quantitative proteomics, bioinformatic analysis, parallel reaction monitoring (PRM), and small RNA interference were used to explore the genes involved in the SCD1-dependent c9, t11-CLA biosynthesis. Through the tandem mass tag (TMT)-based quantitative proteomics and bioinformatic analysis, 60 differentially expressed proteins that were mainly enriched in the pathways of energy metabolism were preliminary screened in this study. Energy metabolism and lipid metabolism are inextricably linked, glycolysis-producing dihydroxyacetone phosphate reduction forms of glycerol, pyruvate oxidative decarboxylation forms acetyl coenzyme A, a raw material for fatty acid biosynthesis [49]. In addition, the pentose phosphate pathway is also closely related to fatty acid formation, and the reductase II (NADPH) produced in the pentose phosphate pathway is used in the biosynthesis of fatty acids [18,50,51,52]. Nucleotides, such as NAD+, NADP+, AD, and CoA, can regulate many biological processes and participate in the biosynthesis of coenzymes. Several important coenzymes of nucleotide coenzymes are adenosine derivatives. NAD+ and FAD can participate in many redox reactions through the transfer of hydrogen atoms, while CoA activates fatty acids, which are related to fatty acid oxidation and steroid biosynthesis [53].

To check the reliability of the proteomics analysis results, a comparison was made between the PRM validation results and the TMT labeling results. In total, 17 proteins were validated using this comparison. The consistency of the PRM validation results and proteomics trends indicates the reliability of this experiment’s results. Then, these 17 proteins underwent individual knockdown by their specific siRNA sequences in MAC-T cells. After the siRNA interference, PGLS, PFKL, ALDOA, TPI1, GAPDH, PGK1, PGAM1, ENO1, PKM, LDHA, and LDHB were verified to have a negative relationship with SCD1. PGLS, a hydrolase, specifically catalyzes the hydrolysis from 6-phosphogluconolactone to 6-phosphogluconic acid [54,55]. PGLS plays an important role in the production of metabolites 5-phosphoribose (R5P) and NADPH in the pentose phosphate pathway [55]. PFKL is the most important rate-limiting enzyme in glycolysis [56,57,58]. ALDOA is a glycolytic enzyme that catalyzes the reversible conversion of fructose-1,6-bisphosphate to glyceraldehyde 3-phosphate and dihydroxyacetone phosphate [59]. TPI1 catalyzes the mutual transformation of dihydroxyacetone phosphate (DHAP) and d-glyceraldehyde-3-phosphate (G3P) during glycolysis and gluconeogenesis. GAPDH catalyzes the phosphorylation and oxidation of glyceraldehyde-3-phosphate at the same time, and NAD+ is used as the electron acceptor to generate 1,3-diphosphoglyceric acid, thus generating NADH [60]. PGK1 plays a role in the glycolytic pathway, catalyzing the transfer of high-energy phosphate from high-energy phosphate from the 1-position of 1,3-biphosphoglycerate (1,3-BPG) to ADP, thus generating of 3-phosphoglycerate (3-PG) and ATP [61]. PGAM1 is a glycolytic enzyme, which can catalyze the mutual transformation of 3-phosphoglycerate (3PG) and 2-phosphoglycerate (2PG) and 2,3-bisphosphoglycerate (2,3-BPG) as a cofactor in glycolysis [62]. As a moonlight protein, ENO1 performs multiple biochemical functions, such as catalyzing the conversion of 2-phosphoglycerate (2-PGA) to phosphoenolpyruvic acid (PEP) in the glycolytic pathway [63]. PKM is a subtype of pyruvate kinase (PK) which is known as the glycolytic enzyme that catalyzes the final step in glycolysis [64]. The enzyme LDH is a major player in the glucose metabolism, which can catalyze pyruvate to lactic acid [65].

These proteins play a key role in the SCD1-dependent gene network of c9, t11-CLA biosynthesis. Through the above analysis and a literature review, the 11 genes that are mainly involved in the energy metabolism pathways found in this study have close links with the SCD1-dependent molecular mechanism of c9, t11-CLA biosynthesis. This study not only provides a theoretical basis and the technical means for further elucidating the molecular mechanism of c9, t11-CLA synthesis in mammary gland of dairy cows, it also has important clinical implications for revealing the individual difference in c9, t11-CLA synthesis ability of dairy cows.

## 5. Conclusions

In total, 60 SCD1-related proteins mainly involved in energy metabolism pathways were screened in MAC-T cells using the proteomics approaches in this study. Additionally, 17 proteins were validated under PRM analysis. Then, PGLS, PFKL, ALDOA, TPI1, GAPDH, PGK1, PGAM1, ENO1, PKM, LDHA, and LDHB were verified to have a negative relationship with SCD1 after the small RNA interference analysis. Subsequently, the SCD1-dependent gene network of c9, t11-CLA biosynthesis was outlined, which provided a fresh understanding of this process from the perspective of energy metabolism.

## Data Availability

Not applicable.

## Supplementary Materials

The following supporting information can be downloaded at: https://www.mdpi.com/article/10.3390/ani12131718/s1, Table S1: Primer sequences for real-time polymerase chain reaction; Table S2: The sequences of scrambled and specific siRNA; Table S3: Reaction system of real-time PCR; Table S4: Fluorescence quantitative reaction procedure; Table S5: The proteins involved in glycolysis pathway screened from the tandem mass tag-based quantitative proteomics analysis; Table S6: The proteins involved in nucleotide catabolism pathway screened from the tandem mass tag-based quantitative proteomics analysis; Table S7: The proteins involved in other energy metabolism pathway screened from the tandem mass tag-based quantitative proteomics analysis; Table S8: The proteins involved in fat metabolism pathway screened from the tandem mass tag-based quantitative proteomics analysis.
