# Supplementary material for: Proteomics Insights into the Gene Network of cis9, trans11-Conjugated Linoleic Acid Biosynthesis in Bovine Mammary Gland Epithelial Cells
Source: Animals (Basel). 2022 Jul 2;12(13):1718. doi: 10.3390/ani12131718 (PMC9264836; doi:10.3390/ani12131718)
Supplement: Supplementary file 1 [file animals-12-01718-s001.zip › animals-1697756-supplementary.pdf]

Supplementary Table S1. Primer sequences for real-time polymerase chain reaction.

Gene name	Accession number	Primer sequence (5′–3′)	References
β-actin	NM_173979.3	F: GCGTGGCTACAGCTTCACC<br>R: TTGATGTCACGGACGATTTC	[23]
SCD1	NM_173959.4	F: CCCTTTCCTTGAGCTGTCTG<br>R: ATGCTGACTCTCTCCCCTGA	
PGLS	NM_001038580.1	F:GTGGTTTCGGAGAGTCACTGATGG<br>R: TTCTGGGTGTGGGTCCTGATGG	
PFKL	NM_001080244.2	F:CCGCTGGATCTCTGTCTTCATCTTC<br>R:CCTGGCTACTGTGACTGGCATTG	
ALDOA	NM_001101915.1	F: GTTGCGTGCTGAAGATCGGG<br>R: CTCCACGATGGGCACAATGC	
TPI1	NM_001013589.3	F: GCAGAGGGACTTGGAGTGAT<br>R: ATAGGCCAAGACAACCTTGC	[23]
GAPDH	NM_001034034.2	F:CCACATACTCAGCACCAGCATCAC<br>R: CGGCACAGTCAAGGCAGAGAAC	
PGK1	NM_001034299.1	F:GCACAGCAAGTAGCAGTGTCTCC<br>R:GCCCTTATGGATGAGGTGGTGAAAG	
PGAM1	NM_001034054.1	F: GGGAAACGGGTACTAATTGC<br>R: GCAGGTTCAGCTCCATGATA	[23]
ENO1	NM_174049.2	F:TGAGAGCCACCGTTGATGACATTG<br>R:CAAGGCTGGTGCTGTGGAGAAG	
PKM	NM_001205727.1	F:CTTACACACCACAGGGAAGATGCC<br>R:GCCATAATCGTCCTCACCAAGTCTG	
LDHA	NM_174099.2	F: TTGGTCCAGCGTAACGTGAACATC<br>R: AAGCCACTTATCTTCCAAGCCACATAG	
LDHB	NM_174100.2	F: CTTCCTCCCTCCTTGCGGAT<br>R: AGAGTTGCCATTGTGCCCAG	

SCD1 stearoyl-CoA desaturase 1, PGLS 6-phosphogluconolactonase, PFKL phosphofructokinase, liver type, ALDOA aldolase, fructose-bisphosphate A, TPI1 triosephosphate isomerase 1, GAPDH glyceraldehyde-3-phosphate dehydrogenase, PGK1 phosphoglycerate kinase 1, PGAM1 phosphoglycerate mutase 1, ENO1 enolase 1, PKM pyruvate kinase M1/2, LDHA lactate dehydrogenase A, LDHB lactate dehydrogenase B.

Supplementary Table S2. The sequences of scrambled and specific siRNA.

Gene name	Specific siRNA sequences		References
	Scramble	CCTACGCCACCAATTTCGT	[20]
PGLS	siRNA1	UCUCCAAGCUCCCUAUCUUTT	
	siRNA2	GCAAGGCAGCUAUUCUGAATT	
	siRNA3	GGAAACUUUGCUGGUUUCUTT	
PFKL	siRNA1	GGUCCCGCCUCAACAUUAUTT	
	siRNA2	GCGGGAGCUUUGAGAACAATT	
	siRNA3	CCACAGAGUUCCUGUACAATT	
ALDOA	siRNA1	CCGAGAACACUGAGGAGAATT	
	siRNA2	CCCAGGAAGAAUACGUCAATT	
	siRNA3	CCUACGCCACCAAUUUCGUTT	
TPI1	siRNA1	GGACUUGGAGUGAUUGCCUTT	
	siRNA2	GCAGAUAACGUGAAGGAUUTT	
	siRNA3	CCUCAAGCCUGAGUUCGUUTT	
GAPDH	siRNA1	GGUCUACAUGUUCCAGUAUTT	
	siRNA2	GCAUCGUGGAGGGACUUAUTT	
	siRNA3	CCAAGUAUGAUGAGAUCAATT	
PGK1	siRNA1	CCAAGUCAGUUGUUCUUAUTT	
	siRNA2	GCUCCAUGGUAGGAGUAAATT	
	siRNA3	CCUGGAAGGUAAAGUGCUUTT	
PGAM1	siRNA1	CCTACGCCACCAATTTCGT	
	siRNA2	GCACATGGAACCTGGAGAA	[20]
	siRNA3	TCTATGAGTTGGACAAGAA	
ENO1	siRNA1	GCGCUUCAACUGGAAUCUATT	
	siRNA2	GCAUCGGAGCAGAGGUUUATT	
	siRNA3	GCUGAAAGUGAACCAGAUUTT	
PKM	siRNA1	GGAAAGAACAUCAAGAUAATT	
	siRNA2	GGAAUGAAUGUGGCUCGUUTT	
	siRNA3	GGGUGAACUUGGCCAUGAATT	
LDHA	siRNA1	GCUGAUUCAGAAUCUUCUUTT	
	siRNA2	GCAAACUCCAGGCUGGUUATT	
	siRNA3	GCCGAUUUGGCAGAAAGUATT	
LDHB	siRNA1	CCUGAAUGCUCGAGGGUUATT	
	siRNA2	GCCAUCAGCAUUCUGGGAATT	
	siRNA3	GCUAGAUUUCGCUACCUUATT	

Supplementary Table S3. Reaction system of real-time PCR.

Component	Volume (μL)
cDNA	2
Forward Primer(10pM/μL)	0.6
Reverse Primer(10pM/μL)	0.6
2×Talent qPCR PreMix	10
50×ROX Reference Dye△	2
RNase-Free ddH2O	4.8
Total	20

Supplementary Table S4. Fluorescence quantitative reaction procedure.

Phase	Cycle	Temperature	Time	Content	Fluorescence signal acquisition
Pre degeneration	1×	95℃	3min	Pre degeneration	No
PCR reaction	40×	95℃	5sec	Transgender	No
		60℃	15sec	Annealing/extension	Yes

Supplementary Table S5. The proteins involved in glycolysis pathway screened from the tandem mass tag-based quantitative proteomics analysis.

Protein accession	Protein description	Gene name
NP_001013607.1	triosephosphate isomerase	TPI1
NP_001019695.2	triokinase/FMN cyclase	TKFC
NP_001029226.1	phosphoglycerate mutase 1	PGAM1
NP_001029874.1	beta-enolase	ENO3
NP_001030213.1	galactose-1-phosphate uridylyltransferase	GALT
NP_001030361.1	mannose-6-phosphate isomerase	MPI
NP_001035642.1	glyceraldehyde-3-phosphate dehydrogenase, testis-specific	GAPDHS
NP_001070371.1	phosphoglucomutase-1	PGM1
NP_001073713.1	ATP-dependent 6-phosphofructokinase, liver type	PFKL
NP_001092851.1	galactokinase	GALK1
NP_001094595.1	gamma-enolase	ENO2
NP_001095385.1	fructose-bisphosphate aldolase A	ALDOA
NP_001116507.1	phosphoglycerate kinase 2	PGK2
NP_001180149.1	ATP-dependent 6-phosphofructokinase, platelet type	PFKP
NP_001193137.1	UDP-glucose 4-epimerase	GALE
NP_776524.1	L-lactate dehydrogenase A chain	LDHA
NP_777237.3	6-phosphofructo-2-kinase/fructose-2,6-bisphosphatase 2	PFKFB2
NP_001091453.1	fructose-bisphosphate aldolase C	ALDOC
NP_001029206.1	glyceraldehyde-3-phosphate dehydrogenase	GAPDH
NP_001029421.1	alcohol dehydrogenase class-3	ADH5
NP_001029471.1	phosphoglycerate kinase 1	PGK1
NP_001029967.1	aldose 1-epimerase	GALM
NP_001069981.1	alcohol dehydrogenase [NADP(+)]	AKR1A1
NP_001192656.1	pyruvate kinase PKM	PKM
NP_776474.2	alpha-enolase	ENO1
NP_776525.2	l-lactate dehydrogenase B chain isoform LDHB	LDHB

Supplementary Table S6. The proteins involved in nucleotide catabolism pathway screened from the tandem mass tag-based quantitative proteomics analysis.

Protein accession	Protein description	Gene name
NP_001013607.1	triosephosphate isomerase	TPI1
NP_001029207.1	hypoxanthine-guanine phosphoribosyltransferase	HPRT1
NP_001029226.1	phosphoglycerate mutase 1	PGAM1
NP_001029874.1	beta-enolase	ENO3
NP_001030213.1	galactose-1-phosphate uridylyltransferase	GALT
NP_001069150.2	cytosolic acyl coenzyme A thioester hydrolase	ACOT7
NP_001069496.1	deoxyribose-phosphate aldolase	DERA
NP_001070371.1	phosphoglucomutase-1	PGM1
NP_001073713.1	ATP-dependent 6-phosphofructokinase, liver type	PFKL
NP_001091453.1	fructose-bisphosphate aldolase C	ALDOC
NP_001094595.1	gamma-enolase	ENO2
NP_001095385.1	fructose-bisphosphate aldolase A	ALDOA
NP_001180149.1	ATP-dependent 6-phosphofructokinase, platelet type	PFKP
NP_001193137.1	UDP-glucose 4-epimerase	GALE
NP_776397.1	xanthine dehydrogenase/oxidase	XDH
NP_776524.1	L-lactate dehydrogenase A chain	LDHA
NP_776830.1	cytosolic purine 5'-nucleotidase	NT5C2
NP_776842.1	cGMP-specific 3',5'-cyclic phosphodiesterase	PDE5A
NP_001007819.1	purine nucleoside phosphorylase	EDIP
NP_001019695.2	triokinase/FMN cyclase	TKFC
NP_001030361.1	mannose-6-phosphate isomerase	MPI
NP_001033291.1	dCTP pyrophosphatase 1	DCTPP1
NP_001075935.1	diphosphoinositol polyphosphate phosphohydrolase 1	NUDT3
NP_001092851.1	galactokinase	GALK1
NP_001116507.1	phosphoglycerate kinase 2	PGK2
NP_777237.3	6-phosphofructo-2-kinase/fructose-2,6-bisphosphatase 2	PFKFB2
NP_787006.1	histidine triad nucleotide-binding protein 1	HINT1
NP_001029486.1	N-acetyl-D-glucosamine kinase	NAGK
NP_001030267.1	phosphomannomutase 2	PMM2
NP_001039869.1	UDP-N-acetylhexosamine pyrophosphorylase	UAP1
NP_001039947.1	sialic acid synthase	NANS
NP_001070351.1	glutamine--fructose-6-phosphate aminotransferase [isomerizing] 2	GFPT2
NP_001073756.2	glucosamine-6-phosphate isomerase 1	GNPDA1
NP_001073800.1	GDP-mannose 4,6 dehydratase	GMDS
NP_001103431.1	glutamine--fructose-6-phosphate aminotransferase [isomerizing] 1	GFPT1
NP_001178072.2	bifunctional UDP-N-acetylglucosamine 2-epimerase/N-acetylmannosamine kinase	GNE
NP_001193104.1	mannose-1-phosphate guanyltransferase alpha	GMPPA
NP_776637.1	UTP-glucose-1-phosphate uridylyltransferase	UGP2

Supplementary Table S7. The proteins involved in other energy metabolism pathway screened from the tandem mass tag-based quantitative proteomics analysis.

Biological processes	Protein accession	Protein description	Gene name
Monosaccharide catabolism	NP_001029874.1	beta-enolase	ENO3
	NP_001030213.1	galactose-1-phosphate uridylyltransferase	GALT
	NP_001035642.1	glyceraldehyde-3-phosphate dehydrogenase, testis-specific	GAPDHS
	NP_001069981.1	alcohol dehydrogenase [NADP(+)]	AKR1A1
	NP_001070371.1	phosphoglucomutase-1	PGM1
	NP_001073713.1	ATP-dependent 6-phosphofructokinase, liver type	PFKL
	NP_001091453.1	fructose-bisphosphate aldolase C	ALDOC
	NP_001091548.1	ribulose-phosphate 3-epimerase	RPE
	NP_001094595.1	gamma-enolase	ENO2
	NP_001180149.1	ATP-dependent 6-phosphofructokinase, platelet type	PFKP
	NP_001193137.1	UDP-glucose 4-epimerase	GALE
	NP_776524.1	l-lactate dehydrogenase A chain	LDHA
Pentose phosphate pathway	NP_001003906.1	transketolase	TKT
	NP_001030360.2	transaldolase	TALDO1
	NP_001033669.1	6-phosphogluconolactonase	PGLS
	NP_001069496.1	deoxyribose-phosphate aldolase	DERA
	NP_001070371.1	phosphoglucomutase-1	PGM1
	NP_001073713.1	ATP-dependent 6-phosphofructokinase, liver type	PFKL
	NP_001091548.1	ribulose-phosphate 3-epimerase	RPE
	NP_001095385.1	fructose-bisphosphate aldolase A	ALDOA
	NP_001107624.1	ribose-phosphate pyrophosphokinase 2	PRPS2
	NP_001137210.1	6-phosphogluconate dehydrogenase, decarboxylating	PGD
	NP_001180149.1	ATP-dependent 6-phosphofructokinase, platelet type	PFKP

Supplementary Table S8. The proteins involved in fat metabolism pathway screened from the tandem mass tag-based quantitative proteomics analysis.

Biological processes	Protein accession	Protein description	Gene name
Lipid-related Acyl-CoA biological processes	NP_001040062.1	elongation of very long chain fatty acids protein 5	ELOVL5
	NP_001095625.1	elongation of very long chain fatty acids protein 6	ELOVL6
	NP_776579.1	palmitoyl-protein thioesterase 1 precursor	PPT1
Lipoprotein catabolism	NP_001160002.1	low-density lipoprotein receptor precursor	LDLR
	NP_776579.1	palmitoyl-protein thioesterase 1 precursor	PPT1
Sphingolipid catabolism	NP_776579.1	palmitoyl-protein thioesterase 1 precursor	PPT1
	NP_001039814.1	alpha-N-acetylgalactosaminidase precursor	NAGA
Fatty acid elongase activity	NP_001040062.1	elongation of very long chain fatty acids protein 5	ELOVL5
	NP_001095625.1	elongation of very long chain fatty acids protein 6	ELOVL6
Fatty acid extension pathway	NP_001040062.1	elongation of very long chain fatty acids protein 5	ELOVL5
	NP_001095625.1	elongation of very long chain fatty acids protein 6	ELOVL6
	NP_776579.1	palmitoyl-protein thioesterase 1 precursor	PPT1
Biosynthesis of UFA	NP_001040062.1	elongation of very long chain fatty acids protein 5	ELOVL5
	NP_001095625.1	elongation of very long chain fatty acids protein 6	ELOVL6
	NP_001096786.1	very-long-chain (3R)-3-hydroxyacyl-CoA dehydratase 3	HACD3
	NP_776384.3	stearoyl-coenzyme A desaturase	SCD1
